# Classification for Single-Trial N170 During Responding to Facial Picture With Emotion

**DOI:** 10.3389/fncom.2018.00068

**Published:** 2018-09-13

**Authors:** Yin Tian, Huiling Zhang, Yu Pang, Jinzhao Lin

**Affiliations:** College of Bio-information, Chongqing University of Posts and Telecommunications, Chongqing, China

**Keywords:** single-trial, N170, facial recognition, emotional classification, BCIs

## Abstract

Whether an event-related potential (ERP), N170, related to facial recognition was modulated by emotion has always been a controversial issue. Some researchers considered the N170 to be independent of emotion, whereas a recent study has shown the opposite view. In the current study, electroencephalogram (EEG) recordings while responding to facial pictures with emotion were utilized to investigate whether the N170 was modulated by emotion. We found that there was a significant difference between ERP trials with positive and negative emotions of around 170 ms at the occipitotemporal electrodes (i.e., N170). Then, we further proposed the application of the single-trial N170 as a feature for the classification of facial emotion, which could avoid the fact that ERPs were obtained by averaging most of the time while ignoring the trial-to-trial variation. In order to find an optimal classifier for emotional classification with single-trial N170 as a feature, three types of classifiers, namely, linear discriminant analysis (LDA), L1-regularized logistic regression (L1LR), and support vector machine with radial basis function (RBF-SVM), were comparatively investigated. The results showed that the single-trial N170 could be used as a classification feature to successfully distinguish positive emotion from negative emotion. L1-regularized logistic regression classifiers showed a good generalization, whereas LDA showed a relatively poor generalization. Moreover, when compared with L1LR, the RBF-SVM required more time to optimize the parameters during the classification, which became an obstacle while applying it to the online operating system of brain-computer interfaces (BCIs). The findings suggested that face-related N170 could be affected by facial expression and that the single-trial N170 could be a biomarker used to monitor the emotional states of subjects for the BCI domain.

## Introduction

Emotion is considered to be a subjective perception or feeling toward the internal and external environment, along with some kind of physiological response (Khushaba et al., 2013). Individuals might suffer from mental disorders, such as anxiety and depression, if they do not correctly handle the mood swings caused by psychological or physical damage over a long period of time (Lindquist and Barrett, 2012). Nowadays, mental illness, that has continuously troubled the society, has become a serious problem; thus, the need to identify measures to prevent and treat such disorders has become a vital project. Recently, the rapid development of neuroscience has facilitated improvements in brain-computer interfaces (BCIs), which enable direct communication with external devices bypassing the usual peripheral neural pathways (Panicker et al., 2011; Mühl et al., 2014). Research in the areas of affective neuroscience and emotion classification in BCIs has become of great value to the fields of emotion monitoring and rehabilitation therapy. Moreover, the search for features representing individual emotions or emotional states based on physiological signals has brought prospects in the application of BCIs to comprehend the harmonious interaction between the brain and computers.

Facial expressions, which might embody a person's emotional state, are often utilized to study emotion and for the classification of facial expressions with emotion in the experimental environment with regard to cognition. A large amount of research has shown that compared to other object categories, the human face elicits a larger negative amplitude waveform at a latency of about 170 ms, which was termed as N170 (Bentin et al., 1996). According to previous studies, the N170 was more likely to be generated at occipito-temporal areas, and it might be involved in face perceptual processing (Song et al., 2017). More surprisingly, several studies found an interesting phenomenon that the N170 components might be affected by facial emotion (Caharel et al., 2005; Liu et al., 2013; Rellecke et al., 2013). Therefore, the research that N170 components were utilized in emotional classification might be valuable for the field of emotional BCIs.

Previous studies showed that compared to spontaneous brain activity based on BCIs such as motor imaginary (Pfurtscheller and Neuper, 2001; Toshiro et al., 2012), the performance of evoked brain activity based on BCIs had a higher signal-to-noise ratio and faster mental state recognition (Meng et al., 2008; Jin et al., 2011) such as event-related potentials (ERPs). For example, P300, an electrophysiological response to a novel internal or external stimulus, was a typical positive peak around 300 ms following the presentation of infrequent target stimulus onset during the oddball paradigm (Fazel-Rezai et al., 2012). Similarly, the motion-specific N200 ERPs produced a negative peak around 200 ms after stimuli onset, and they were also introduced to an asynchronous BCI speller (Hong et al., 2009; Zhang D. et al., 2012). In fact, compared with N200 and P300 ERP usually used in the field of BCIs, N170 ERP provides a faster and early processing of brain components in the time course, which might contribute to the real time research of BCIs. However, N170 was used as a feature for facial classification by most of the previous studies (faces vs. non-faces), but it was not used for pattern classification in relation to emotional recognition (Zhang Y. et al., 2012; Cai et al., 2013).

Moreover, an increasing number of features extracted from electroencephalogram (EEG) signals, such as EEG time-frequency features (Chanel et al., 2009), boosting encoded dynamic features (Yang et al., 2009), recoursing energy efficiency (REE), and root mean square (RMS) (Murugappan et al., 2008), were used to classify emotion. However, the classification features used in these studies were generally based on averaged trial ERPs from EEG recordings, but were not based on single-trial ERPs, which might lead to the trial-to-trial variation being neglected.

Therefore, we proposed single-trial N170 ERPs elicited by the facial pictures with different emotions to apply for emotional classification, and we focused on the linear/nonlinear characteristics of the chosen classifiers to obtain the generalization performance such as classification accuracy, sensitivity, computational time, and so on. Thus, a comparative study was performed by using three types of classifiers, namely, linear discriminant analysis (LDA), L1-regularized logistic regression (L1LR), and support vector machine with radial basis function (RBF-SVM). LDA represented a classical linear classifier, L1LR represented a special case of a generalized linear model, and RBF-SVM represented a classical nonlinear classifier. Moreover, the three types of classifiers were easy to implement. Therefore, the three classifiers were utilized to reveal the potential relationship between positive and negative emotions in the current study.

We assumed that the single-trial N170 ERP could be used as a feature to successfully classify positive and negative emotions, and we intended to find an optimal classifier for emotional classification. These findings might provide a meaningful reference for the development of emotional classification and emotion regulation in BCIs.

## Materials and methods

### Ethics statement

Informed consent was signed prior to the study, and subjects also received monetary compensation after the experiments. All experiments were approved by the ethical committee of Chongqing university of Posts and Telecommunications. All experimental procedures were conducted in accordance with the ethical guidelines determined by the National Ministry of Health, Labor and Welfare and the Declaration of Helsinki (BMJ 1991; 302:1194).

### Subjects

Twenty healthy and right-handed subjects (male: 10; female: 10; mean age: 21 years) from the EEG Laboratory of Chongqing University of Posts and Telecommunications participated in the experiment. None of the subjects had cognitive impairments or mental or neurological disorders. All subjects' vision or corrected vision was normal. The grade point average (GPA) of the subjects was shown in Appendix 1 (Supplementary Material). The experiment consisted of four blocks of 120 trials each (480 trials in total with 160 trials × 3 emotions). Moreover, a pseudorandom approach was adopted to prevent stimulus repetition. Subjects were required to maintain central fixation and minimize eye blinks and body motion throughout the recordings. Stimuli were presented and behavioral data were recorded using E-prime software (http://www.pstnet.com/eprime.cfm).

### Stimuli and design

**Figure 1:**
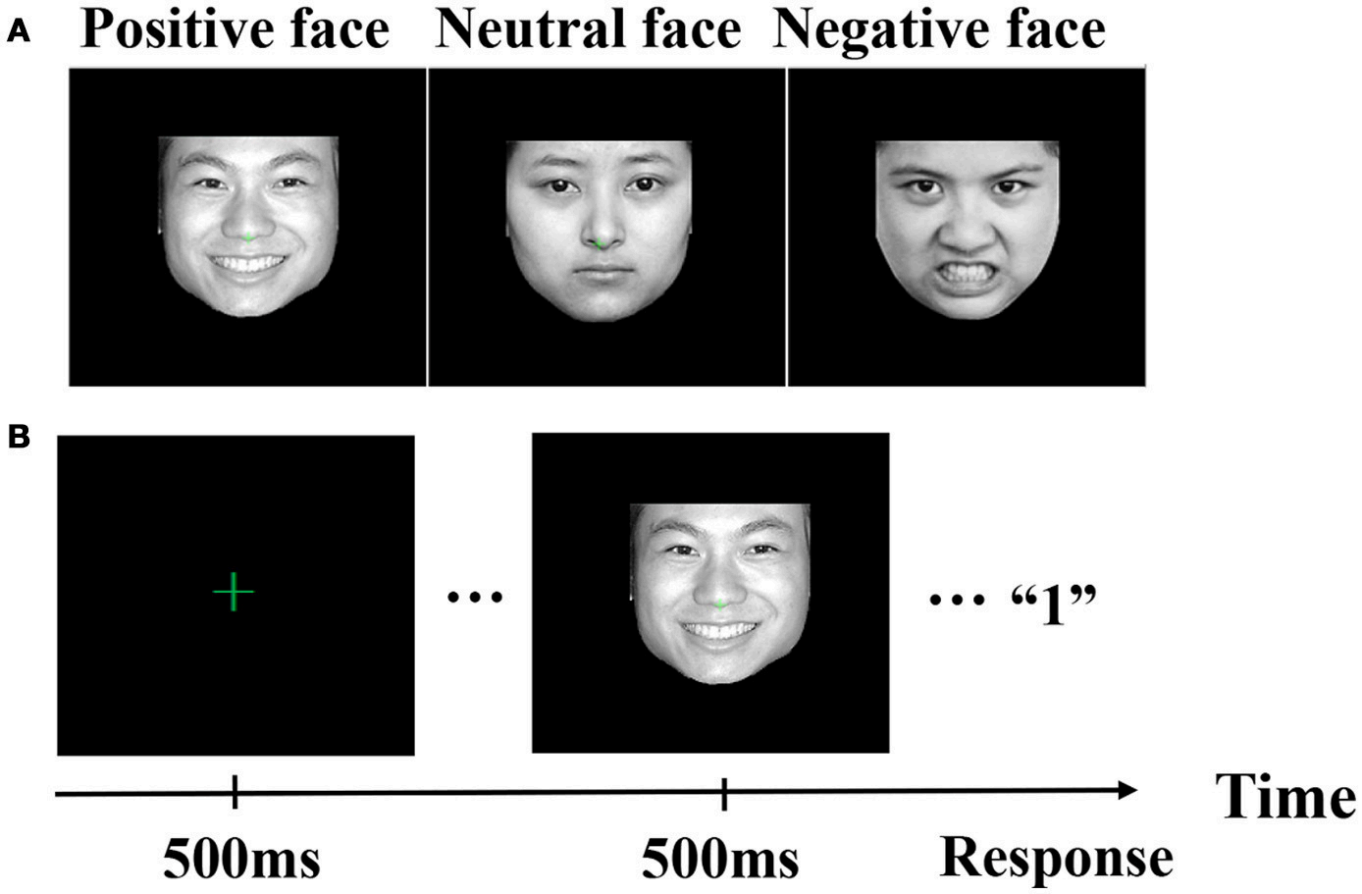
Illustration of facial stimuli. **(A)** Facial stimuli with three different emotions. **(B)** An example of the stimulus sequence with emotional pictures.

Figure 1A showed facial stimuli with three different emotions. Moreover, stimuli were photographic faces without external characteristics. Usually, positive emotion denoted happy, joyful, smiles and laughs. Negative emotion denoted sad, terrified, and jealous, and neutral emotion was expressionless. Emotion valence and arousal of facial stimuli were shown in Appendix 1 (Supplementary Material). Figure 1B illustrated an example of the stimulus sequence with emotional pictures. A green fixation cross (0.5 × 0.5; at the center of the monitor) was displayed throughout the entire block of trials, and the visual angle displayed by the photos was 4 × 4. Each trial started with the fixation cross flashing for 500 ms. Following that, one of three expression pictures (i.e., positive, neutral, and negative) was presented for 500 ms and subjects were asked to respond to discriminate expressions (positive, neutral, and negative) with a button press using their right hand (index finger, middle finger, and ring finger). In other words, subjects needed to press key “1” using their right index finger if a positive face appeared, subjects needed to press key “2” using their right middle finger if a neutral face appeared, and subjects needed to press key “3” using their right ring finger if a negative face appeared. If subjects did not make a timely response within the maximum allowable response time (1,200 ms), the next stimulus sequence would be represented. Response accuracy and speed were equally emphasized in the instructions. All 480 pictures were randomly presented in a mixed design, with four blocks of 120 stimuli each. For each subject, there were 160 trials for each emotion (i.e., positive: 160 trials, neutral: 160 trials, and negative: 160 trials).

### Behavioral analysis

Subjects' response accuracy (ACC) and reaction time (RT) were recorded and analyzed using a one-way analysis of variance (ANOVA), with emotion (positive, neutral, and negative) as the within-subjects factor. For each subject, incorrect responses or responses with RT more than mean ± 2SD in each emotion were removed from RT analysis (Liu et al., 2013).

### EEG recording and processing

Electroencephalogram was recorded by a 64-channel NeuroScan system (Quik-Cap, band pass: 0.05–100 Hz, sampling rate: 250 Hz, impedances < 5 kΩ) with a vertex reference. To monitor ocular movements and eye blinks, electrooculogram (EOG) signals were simultaneously recorded from four surface electrodes, with one pair placed over the upper and lower eyelid and the other pair placed 1 cm lateral to the outer corner of the left and right orbit.

The data were re-referenced by the reference electrode standardization technique (REST) (Yao, 2001; Tian and Yao, 2013) which denoted the infinity zero reference. In the study, EEG was segmented from 200 ms before the stimulus onset to 280 ms after the stimulus onset. Electromyography (EMG) and EOG were excluded by blind source separation (BSS) (Negro et al., 2016) and other noise was removed by automatic artifact rejection (±100 μ*V*). The data were baseline corrected using the 200 ms before the stimulus onset, and then the EEG recordings were filtered with a band-pass of 0.5–45 Hz.

### Feature extraction

In the current study, single-trial N170 ERPs were chosen as classification features to classify positive and negative emotions. According to previous studies, N170 was elicited at the occipitotemporal electrodes (Bentin et al., 1996; Cai et al., 2013; Song et al., 2017). Therefore, our focus was on twelve electrodes in occipitotemporal areas: that is, P3, P4, P5, P6, P7, P8, PO3, PO4, PO5, PO6, PO7, and PO8 (Smith et al., 2012). Then, among the twelve electrodes, the paired *t*-test was performed to select the top five channels where obvious differences between positive and negative emotional N170 trials were observed based on the following classification. At the same time, dimensionality reduction was achieved. For each subject, the features were normalized by scaling between 0 and 1 to reduce individual differences (Lin et al., 2008; Chanel et al., 2009).

### Classification

Three types of classifiers were utilized to classify the positive-related and negative-related N170 trials: namely, LDA, L1LR, and RBF-SVM. Moreover, the positive-related N170 ERPs and the negative-related N170 ERPs were defined as positive and negative in the classification process, respectively.

#### Linear discriminant analysis (LDA)

A classical linear classifier (LDA) was proposed by Fisher for binary classification and was, therefore, called Fisher's linear discriminant. Linear discriminant analysis was widely used in face recognition and machine learning to find a linear projection of features that characterized or separated two or more classes of objects or events (Sharma and Paliwal, 2015). The purpose of projecting the labeled training data was to maximize the distance between the two classes' means and minimize the interclass variance (Müller et al., 2003).

#### L1-regularized logistic regression (L1LR)

Logistic regression (LR), as a special case of a generalized linear model (Cook and Weisberg, 2008), was considered to be an alternative to LDA. Logistic regression, a crucial method for statistical analysis, was widely used in various fields, and it had a good effect on practical applications (Keating and Cherry, 2004; Ayalew and Yamagishi, 2005), especially in pattern classification (Zhu and Hastie, 2004).

However, there were some obvious shortcomings in the traditional LR model, mainly in the following two aspects: the selection of variables and overfitting problems. Most of the model parameters fitted by the LR model were not zero, that is, the model was related to most of the variables; therefore, it was not sparse. In effect, in many practical problems, if the model was not sparse, the computational complexity would increase, meaning that the interpretation of practical problems was more difficult. With regard to overfitting problems (Kim et al., 2007), a LR model for the training data could often get a good fit accuracy, but for the test data, the classification accuracy was not ideal.

Therefore, some researchers proposed the L1LR model to overcome the above problems (Kim et al., 2007; Park and Hastie, 2007). The L1LR problem was

(1) $$\mathrm{minimize}\;l_{avg}\left(v,w\right)+\lambda {\left\|w\right\|}_{1}$$

where *l*_*avg*_ was the average logistic loss function; ν∈*R* (the intercept) and *w*∈*R* (the weight vector) were the parameters of the logistic model; and λ > 0 was the regularization parameter, and it was used to control the trade-off between the average logistic loss and the size of the weight vector, as measured by the L1-norm [refer Kim et al. (2007) for more detail].

#### Support vector machine (SVM)

Support vector machine was developed by Vapnik based on statistical learning theory (SLT) (Netherlands, 2008). As a result of its excellent generalization performance, SVM has been applied to a wide variety of issues, such as text classification, images classification, hand writing recognition, and gene classification. Furthermore, SVM had the feature of empirical risk minimization (ERM) and global optimum solution (Netherlands, 2008). Using kernel function, SVM could efficiently perform linear and nonlinear classification by projecting original features into high dimensional feature spaces, which made the two classes easy to distinguish. In the current study, the SVM classification framework was implemented by using the following equation:

(2) $$\begin{array}{l} f\left(x\right)=sign\left(\sum_{i=1}^{n}\beta_{i}y_{i}K\left(x,x_{i}\right)+b\right) \end{array}$$

where *f*(*x*) was the decision function; *n* was the number of trials; β_*i*_∈*R* was the Lagrangian multiplier; *y*_*i*_ denoted 1 or −1, which was the class label; *b* was the bias; and *K*(*x, x*_*i*_) denoted the kernel function. In the current study, we chose radial basis function as the SVM kernel (Brew, 2016).

### Generalization of classifier

If a classifier could predict the class of a new sample with good performance, it was considered to clearly reflect the relationship between the feature and the class label. Besides choosing a reliable feature to represent emotion, the selection of an appropriate classifier was also a critical problem in the field of BCIs. For most of the previous studies, the generalization of classifier was measured just by classification accuracy (CA), which might not be able to effectively evaluate the generalization of a classifier (Jin and Ling, 2005). Therefore, in the current study, combined with 10-fold cross-validation, six types of metrics were utilized to fully evaluate the generalization of the three classifiers. The processing procedure of the 10-fold cross-validation is described below.

Firstly, the initial sample was randomly divided into ten subsamples. Secondly, nine of the ten subsamples were considered to be the training set to establish the SVM model, and the remaining one was retained as the test set to evaluate the generalization of the classifiers. The manipulation was repeated until each subsample had been assigned as a test set on one occasion. Finally, the ten classification results were averaged to obtain the eventual classification results. Six types of metrics for the generalization of the three classifiers were defined by the following expressions.

#### Classification accuracy (CA)

Classification accuracy was defined as the percentage of the number of samples predicted correctly in the test set divided by the total number of the samples, and it was calculated by the following equation:

(3) $$\begin{array}{l} CA=\frac{TP+TN}{TP+TN+FP+FN} \end{array}$$

where true positive (*TP*) was the number of positive samples correctly predicted; true negative (*TN*) was the number of negative samples correctly predicted; false positive (*FP*) denoted the number of incorrectly predicted positive samples; and false negative (*FN*) denoted the number of incorrectly predicted negative samples.

#### Sensitivity (SE) and specificity (SP)

Sensitivity and specificity were calculated by the following formulae, respectively:

(4) $$\begin{array}{l} SE=\frac{TP}{TP+FN} \end{array}$$

(5) $$\begin{array}{l} SP=\frac{TN}{TN+FP} \end{array}$$

Sensitivity referred to the ratio of correctly classified positive samples to the total population of positive samples, whereas *SP* was the ratio of correctly classified negative samples to the total population of negative samples.

#### Area under the curve (AUC)

The AUC was defined as the area under the receiver operating characteristic (ROC) curves, which was discovered and proved to be better than CA to evaluate the predictive performance of classification learning algorithms (Jin and Ling, 2005). Moreover, AUC was indeed a statistically consistent and more discriminating measure than CA (Ling et al., 2003). Originally, only the ROC curves were introduced to evaluate machine learning algorithms (Provost et al., 1997). In the ROC curves, TP was plotted on the Y axis and FP was plotted on the X axis. It described the classifiers' performance across the entire range independent of class distributions (Provost et al., 1997; Jin and Ling, 2005). However, there was often no clear dominating relation between two ROC curves in the entire range. Therefore, AUC was introduced to provide a good “summary” for the performance of the learning algorithms based on ROC.

#### Kappa

(6) $$\begin{array}{l} Kappa=\frac{P\left(O\right)-P\left(E\right)}{1-P\left(E\right)} \end{array}$$

where *P(O)* denoted an observational probability of agreement, and *P(E)* was the hypothetical probability of expected agreement by chance. The Kappa coefficient value ranged from −1 to 1. A Kappa value with 1 denoted a perfect classification, −1 meant a completely incorrect classification, and 0 denoted that the performance of a classifier was equal to a random guess (Landis and Koch, 1977; Eugenio and Glass, 2015).

#### Computational time (CT)

For the application of BCIs, one of the challenges was the real time online processing of signals, which required the classifier to have a good speed of operation. Therefore, the training and testing time, called the CT in the current study, were recorded as the metrics for the classification generalization. All of the runtime experiments were conducted on a personal computer (PC) with Intel® Core™ i7-3770 CPU @ 3.40 GHz, 8 GB RAM.

## Results

### Behavioral performance

**Figure 2:**
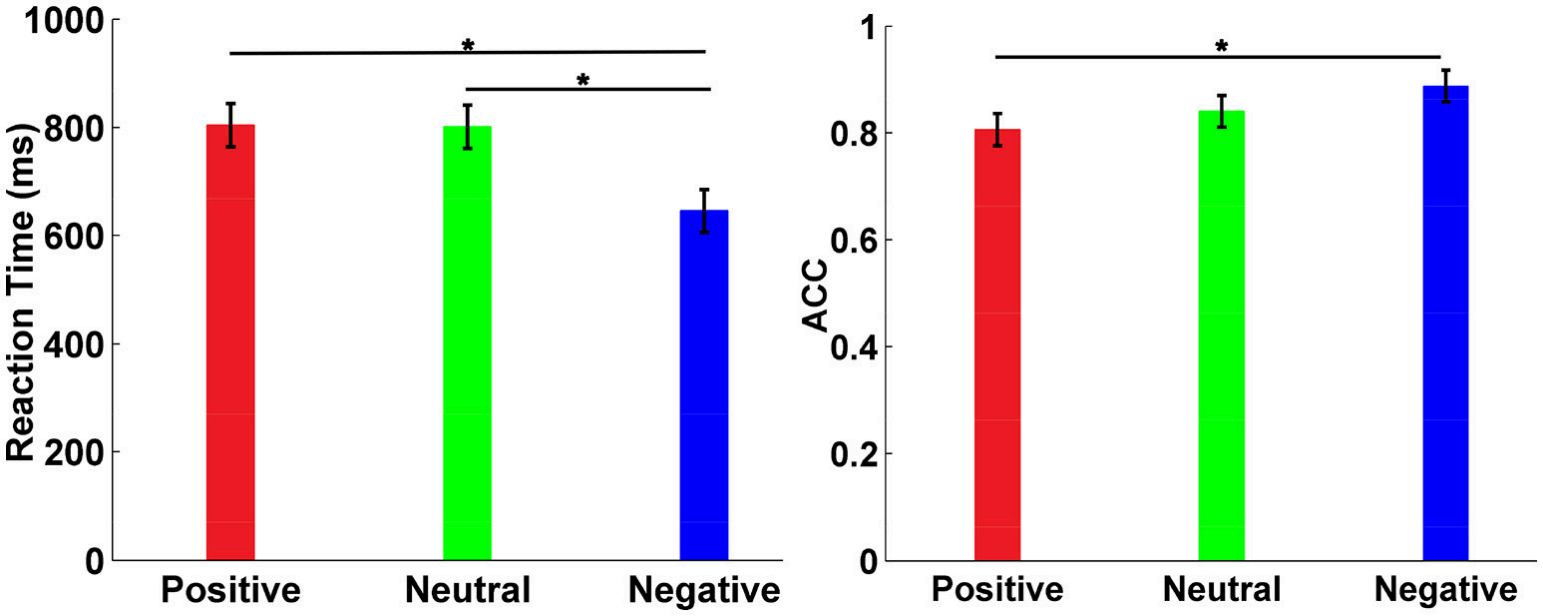
Behavioral performance analysis. **(A)** Mean RT with SD. **(B)** ACC with different emotions. The star denoted that there was a significant difference on RT or ACC between two facial emotions.

Mean RT was shown in Figure 2A with standard deviation (SD). The results displayed a significant main effect of emotion (*F* = 6.28, *p* = 0.003). *Post hoc* test showed that the RT for negative emotion was clearly faster than the RT for positive emotion (*t* = 6.06, *p* < 0.001) and the RT for neutral emotion (*t* = 6.23, *p* < 0.001). However, there was no significant difference between the RT for positive emotion and the RT for neutral emotion (*t* = 0.16, *p* > 0.05). The ACC was analyzed by using the same statistical model as that used for RT, as shown in Figure 2B. There was a significant main effect of emotion on ACC (*F* = 6.28, *p* < 0.05). Moreover, the *post hoc* test showed that the negative faces were identified more correctly than the positive faces (*t* = 3.11, *p* < 0.05), whereas there was no significant difference on ACC between neutral faces identification and negative faces identification (*t* = 1.66, *p* > 0.05). Furthermore, there was no significant difference on ACC between neutral faces identification and positive faces identification (*t* = 1.46, *p* > 0.05).

### ERP analysis

To elaborate the feature extraction window, the ERP waveforms with different emotions were drawn to find the data segments where there were obvious differences between positive and negative emotional N170 ERPs. Positive and negative emotions were tested by the paired *t*-test. The feature extraction window was located on the data segments with significant differences between the positive and negative trials.

**Figure 3:**
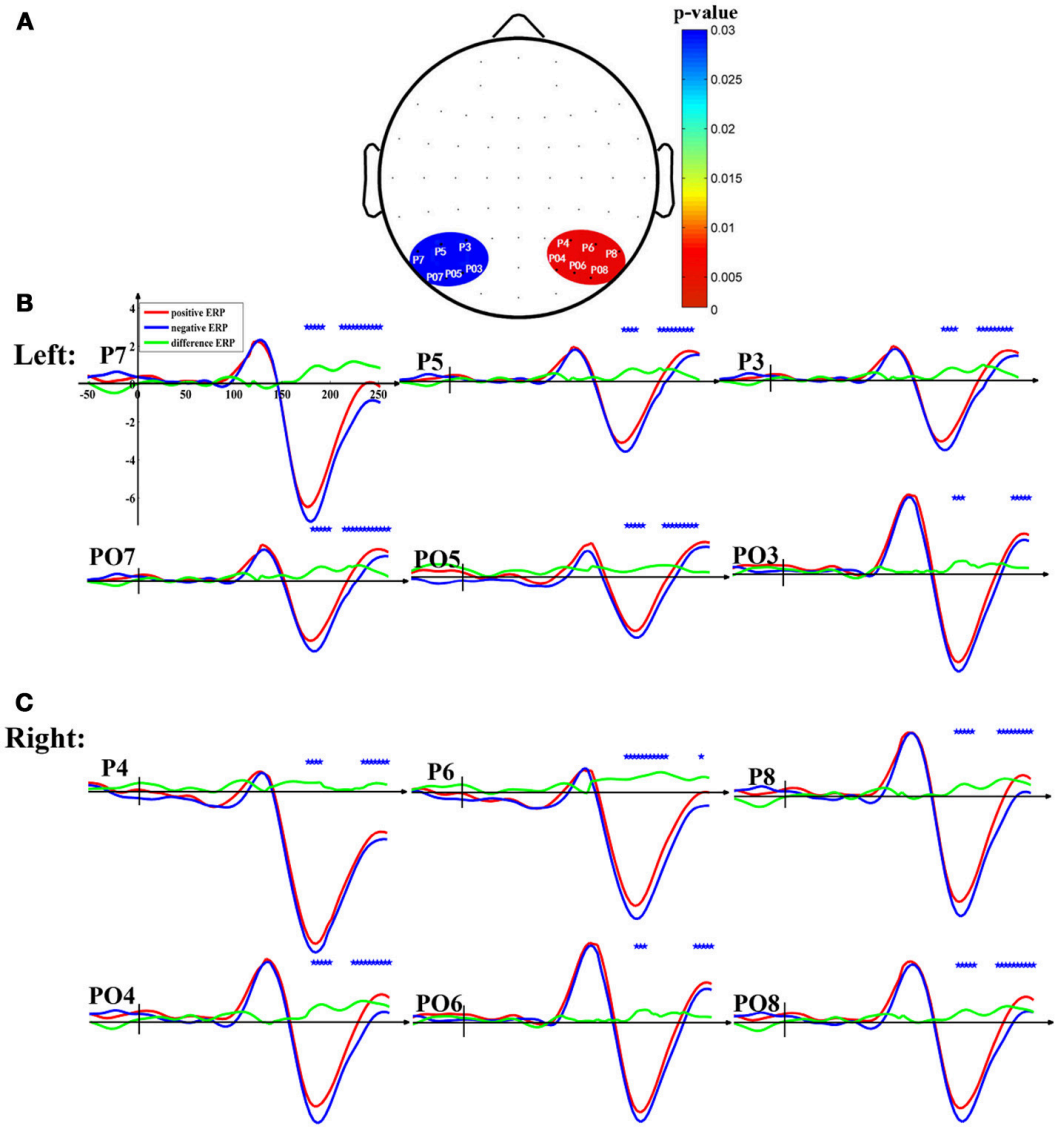
The N170 waveforms elicited by facial pictures with positive and negative emotion. **(A)** Statistical parametric scalp mapping (positive vs. negative). The color bar denoted *p*-values after performing paired *t*-test between positive and negative N170. **(B)** N170 at the left occipitotemporal electrodes. **(C)** N170 at the right occipitotemporal electrodes. The red lines denoted the N170 waveforms elicited by positive faces, the blue lines denoted the N170 waveforms elicited by negative faces, and the green lines were averaged difference-ERP. The blue star denoted that there was a significant difference between positive and negative N170.

As shown in Figure 3, there was a significant difference between positive emotion and negative emotion around 170 ms at all the twelve electrodes (P3, P4, P5, P6, P7, P8, PO3, PO4, PO5, PO6, PO7, and PO8). Moreover, the topographic map illustrated the corresponding *p*-values at the twelve electrodes after performing the paired *t*-test between positive N170 and negative N170 (Figure 3A).

### Generalization for classifier

The classification results of the three classifiers were shown in the tables (L1LR: Table 1; RBF-SVM: Table 2; LDA: Table 3). The generalizations of the three classifiers (L1LR, RBF-SVM, and LDA) were evaluated by six different aspects including CA, AUC, SE, SP, Kappa, and CT.

**Table 1:** Generalization of L1LR.

**L1LR**	**CA**	**AUC**	**SE**	**SP**	**Kappa**	**CT**
S1	0.871	0.887	0.842	0.821	0.680	0.026
S2	0.842	0.882	0.914	0.757	0.675	0.030
S3	0.841	0.901	0.909	0.760	0.656	0.032
S4	0.889	0.887	0.884	0.826	0.685	0.027
S5	0.862	0.898	0.964	0.716	0.705	0.030
S6	0.917	0.978	0.878	0.918	0.784	0.035
S7	0.816	0.934	0.797	0.882	0.645	0.030
S8	0.861	0.941	0.833	0.880	0.717	0.034
S9	0.895	0.914	0.872	0.953	0.717	0.023
S10	0.864	0.941	0.809	0.934	0.717	0.029
S11	0.855	0.935	0.888	0.828	0.698	0.033
S12	0.851	0.950	0.851	0.930	0.712	0.040
S13	0.836	0.831	0.825	0.837	0.629	0.029
S14	0.880	0.866	0.877	0.885	0.763	0.029
S15	0.870	0.886	0.882	0.846	0.718	0.034
S16	0.857	0.868	0.821	0.832	0.720	0.019
S17	0.882	0.886	0.863	0.822	0.705	0.087
S18	0.872	0.90	0.905	0.805	0.705	0.037
S19	0.862	0.890	0.895	0.842	0.714	0.031
S20	0.859	0.879	0.830	0.930	0.704	0.031
Average	0.864 ± 0.022	0.903 ± 0.034	0.867 ± 0.041	0.850 ± 0.064	0.703 ± 0.036	0.033 ± 0.014

*CA, classification accuracy; AUC, area under ROC curves; SE, sensitivity; SP, specificity; CT, computational time*.

**Table 2:** Generalization of RBF-SVM.

**RBF-SVM**	**CA**	**AUC**	**SE**	**SP**	**Kappa**	**CT**
S1	0.816	0.868	0.934	0.736	0.715	102.647
S2	0.774	0.815	0.856	0.682	0.654	111.540
S3	0.819	0.860	0.860	0.781	0.666	107.877
S4	0.809	0.895	0.806	0.803	0.607	106.313
S5	0.840	0.833	0.853	0.835	0.698	101.316
S6	0.870	0.948	0.871	0.874	0.748	97.352
S7	0.848	0.858	0.812	0.814	0.708	92.272
S8	0.842	0.913	0.864	0.860	0.673	112.113
S9	0.866	0.849	0.867	0.875	0.725	86.093
S10	0.875	0.932	0.871	0.875	0.742	102.176
S11	0.858	0.911	0.798	0.879	0.707	109.576
S12	0.847	0.942	0.794	0.881	0.672	99.527
S13	0.791	0.840	0.788	0.809	0.645	109.510
S14	0.823	0.834	0.846	0.810	0.676	104.746
S15	0.848	0.877	0.897	0.827	0.693	87.300
S16	0.889	0.864	0.802	0.787	0.776	57.060
S17	0.855	0.831	0.919	0.817	0.704	103.555
S18	0.850	0.874	0.803	0.861	0.730	94.854
S19	0.867	0.880	0.850	0.825	0.732	95.346
S20	0.875	0.882	0.866	0.887	0.783	90.069
Average	0.843 ± 0.030	0.875 ± 0.038	0.848 ± 0.041	0.826 ± 0.053	0.703 ± 0.044	98.562 ± 12.531

*CA, classification accuracy; AUC, area under ROC curves; SE, sensitivity; SP, specificity; CT, computational time*.

**Table 3:** Generalization of LDA.

**LDA**	**CA**	**AUC**	**SE**	**SP**	**Kappa**	**CT**
S1	0.773	0.902	0.808	0.751	0.550	0.100
S2	0.753	0.874	0.759	0.746	0.503	0.104
S3	0.900	0.954	0.949	0.860	0.800	0.125
S4	0.800	0.864	0.741	0.859	0.594	0.137
S5	0.823	0.958	0.929	0.841	0.770	0.122
S6	0.876	0.951	0.852	0.816	0.736	0.057
S7	0.820	0.899	0.823	0.828	0.632	0.114
S8	0.840	0.935	0.805	0.887	0.678	0.107
S9	0.817	0.820	0.921	0.731	0.619	0.124
S10	0.880	0.941	0.853	0.903	0.750	0.105
S11	0.831	0.925	0.853	0.839	0.652	0.129
S12	0.823	0.894	0.853	0.749	0.632	0.291
S13	0.786	0.790	0.897	0.751	0.571	0.469
S14	0.825	0.865	0.786	0.760	0.628	0.359
S15	0.863	0.907	0.868	0.867	0.716	0.313
S16	0.846	0.874	0.837	0.788	0.695	0.339
S17	0.800	0.840	0.895	0.795	0.606	1.069
S18	0.863	0.934	0.833	0.869	0.714	0.372
S19	0.831	0.847	0.851	0.742	0.662	0.371
S20	0.843	0.854	0.823	0.867	0.735	0.360
Average	0.830 ± 0.037	0.891 ± 0.048	0.847 ± 0.054	0.812 ± 0.056	0.662 ± 0.078	0.258 ± 0.230

*CA, classification accuracy; AUC, area under ROC curves; SE, sensitivity; SP, specificity; CT, computational time*.

According to the classification results mentioned above, the paired *t*-test was conducted to find the obvious differences on the six metrics of generalization among the three classifiers. For CA, the results showed that the L1LR classifier was obviously superior to the RBF-SVM (*t* = 2.966, *p* < 0.01) and the LDA classifiers (*t* = 3.860, *p* < 0.001). Moreover, the RBF-SVM classifier was obviously superior to the LDA classifier (*t* = 1.967, *p* < 0.05). For AUC, the results showed that the L1LR classifier was significantly superior to the RBF-SVM (*t* = 4.670, *p* < 0.001) and the LDA classifiers (*t* = 3.508, *p* < 0.01). However, there was no obvious difference between RBF-SVM and LDA (*t* = 1.560, *p* > 0.05) on AUC. For SE, the results showed that there was no obvious difference between any two of the three classifiers. For SP, the results showed that the L1LR classifier was significantly superior to the RBF-SVM (*t* = 2.081, *p* < 0.05) and the LDA classifiers (*t* = 1.940, *p* < 0.05), while there was no obvious difference between RBF-SVM and LDA (*t* = 0.989, *p* > 0.05). For Kappa, the results showed that L1LR was significantly superior to LDA (*t* = 2.411, *p* < 0.05), while there was no obvious difference between L1LR and RBF-SVM (*t* = 0.026, *p* > 0.05). Furthermore, RBF-SVM significantly overmatched LDA (*t* = 2.575, *p* < 0.01) on Kappa. For CT, the results showed that the computing speed of the L1LR classifier was significantly faster than that of the RBF-SVM (*t* = 35.173, *p* < 0.001) and the LDA classifiers (*t* = 4.595, *p* < 0.001). Moreover, the computing speed of LDA was obviously faster than that of RBF-SVM (*t* = 35.019, *p* < 0.001).

## Discussion

In the present study, a novel feature extracted from the single-trail N170, evoked by facial pictures with emotion, was proposed to classify positive and negative emotions. Combined with the 10-fold cross-validation, six types of metrics (i.e., CA, AUC, SE, SP, Kappa, and CT) were used to evaluate the generalization of the three classifiers (i.e., L1LR, RBF-SVM, and LDA). We found that (1) the N170 at the occipitotemporal electrodes was modulated by facial emotion when REST re-reference was applied; (2) the single-trial N170 could be used as a classification feature to differentiate positive emotion from negative emotion; and (3) compared with the other two classifiers (RBF-SVM and LDA), L1LR showed a good generalization for emotional classification with a single-trial N170 as a feature. The findings could open up a new avenue for monitoring people's mood swings and developing effective BCIs on the regulation of individual emotion.

### Emotion and behavioral response

The RT results showed that negative faces were clearly recognized more quickly than positive and neutral faces. Moreover, the ANOVA analysis showed that ACC for negative faces was significantly superior to ACC for positive faces, indicating that negative faces were identified more effectively than positive faces. Previous studies had also reached similar conclusions that the detection of negative facial emotion was faster and more efficient than the detection of positive emotion (Fox et al., 2000; Schupp et al., 2004).

### N170 and facial emotion with rest reference

Whether facial recognition-related N170 was modulated by facial emotion has always been a controversial topic. Initially, some researchers argued that the N170, the processing of faces, was independent and parallel to that of emotional expression (Caharel et al., 2005; Eimer and Holmes, 2007). However, recent findings suggested that the amplitude of the N170 could be affected by facial expressions (Liu et al., 2013; Song et al., 2017). For instance, neutral expressions elicited smaller N170 amplitudes than other emotional expressions (Blau et al., 2007), and happy faces elicited smaller amplitudes than other emotional expressions (Liu et al., 2013). These diversities of experimental findings might be correlated with differences in design and stimuli during the cognitive experiment. Moreover, some researchers suggested that the effects of emotional modulation on N170 were related to the reference electrodes (Hinojosa et al., 2015). For example, compared with the mastoid reference, common average reference reinforced the emotional modulation effects at the occipitotemporal electrodes where the N170 ERPs typically occurred (Rellecke et al., 2013; Hinojosa et al., 2015). Moreover, our recent study also revealed that the reference technique might play a crucial role in ERPs data interpretation, and we found that REST reference would be a superior choice for precise evaluation of the scalp spatiotemporal changes connected to various cognitive events (Tian et al., 2018). In the current study, we found that N170 could be modulated by emotion when ERPs were re-referenced by REST (Yao, 2001; Tian and Yao, 2013), which supported the opinions that the processing of facial recognition and expression were integrated mechanisms rather than segregated mechanisms (Hinojosa et al., 2015; Song et al., 2017), and that the effects of emotional modulation on facial recognition might be associated with the reference technique (Rellecke et al., 2013; Hinojosa et al., 2015). The details of REST reference were shown in Appendix 2 (Supplementary Material).

### Emotional classification in BCIs

Previously, some researchers were devoted to classifying different emotions with various features. For instance, the three EEG time-frequency features, namely short time Fourier transform (STFT) features, mutual information (MI) features, and peripheral features, were utilized to distinguish different emotions elicited by imagination or recall of different emotional events (Chanel et al., 2009). Some researchers also attempted to use the boosting encoded dynamic features for facial expression recognition (Yang et al., 2009). Moreover, fractal dimension values of the real time EEG were also proposed to be features in BCIs-based emotional classification for music therapy (Sourina et al., 2012).

However, the classification features used in these studies came from the averaged ERPs but not from the single-trial ERPs, which might neglect trial-to-trial difference. Moreover, the generalization of classifier was evaluated just in terms of CA for most of the previous studies, which might not be able to comprehensively evaluate the generalization of a classifier.

Furthermore, in comparison with the BCIs based on spontaneous brain activity like motor imaginary (Pfurtscheller and Neuper, 2001; Toshiro et al., 2012), the performance of the BCIs based on evoked brain activity like P300 (Jin et al., 2011) had some clear strengths, such as higher signal-to-noise ratio and faster mental state recognition (Meng et al., 2008). These types of BCIs, based on evoked brain activity, were dependent of external stimulation, such as facial pictures with different emotion used in the current study.

Therefore, in the current study, the evoked brain activity, namely single-trial N170 ERPs, was utilized for BCI-based emotion classification, which was of great significance for recognizing and steadily monitoring individual emotional states. Furthermore, six types of metrics were utilized to roundly evaluate the generalization of the three classifiers.

Our results demonstrated that differences between positive N170 trials and negative N170 trials existed, which indicate that single-trial N170 could be applied to emotional classification. Based on the analysis of the paired *t*-test from the “RESULTS” section, the three classifiers were ranked according to their classification generalization (Table 4). As shown in Tables 1–3, all the three classifiers demonstrated a good classification effect, but L1LR performed best for pattern classification between positive and negative emotional data in accordance with the rankings (Table 4). Classification accuracy, a commonly used evaluation metric of a classifier, denoted the percentage of the number of samples predicted correctly divided by the total number of samples. For CA, L1LR model showed a good performance, but the CA was just a whole metric of a classifier, which might no longer have good performance when the ratios of positives and negatives changed. Therefore, AUC was introduced to evaluate the generalization of the classifiers. Compared with CA, AUC was independent of changes in class distribution and made full use of the predicted probability value during the classification (Jin and Ling, 2005). As illustrated by Table 4, L1LR was obviously superior to RBF-SVM and LDA in terms of AUC, whereas there was no clear difference in SE among the three classifiers. Sensitivty denoted the probability that positive samples were predicted correctly, indicating that the three classifiers had a similar performance on the prediction of positive samples. However, in terms of SP, which denoted the probability with which the negative samples predict correctly, the L1LR classifier was prominently superior to the RBF-SVM and the LDA classifier. Kappa statistics was the proportion of correctly classied samples after accounting for the probability of chance level in the current study. The larger the Kappa value, the better the performance of the classifier. For Kappa, the results showed that L1LR and RBF-SVM were significantly superior to LDA. In the field of BCIs, one of the most difficult challenges was the real time online processing of signals, which required the classifier to have a good speed of operation. Therefore, CT was used as one of the evaluation metrics for the classification generalization in the current study. With respect to CT, the computational time of L1LR was significantly lower than that of RBF-SVM and LDA. In comparison with L1LR, the most significant drawback of RBF-SVM was time-consuming. RBF-SVM created complex nonlinear boundaries, depending on the RBF kernel function used in the current study; that is, more time was required to optimize the parameters during the classification, making it difficult to apply for BCIs via an online operating system.

**Table 4:** Winner of three classifiers.

	**CA**	**AUC**	**SE**	**SP**	**Kappa**	**CT**	**Winner**
NO.1	L1LR	L1LR	n.s.	L1LR	L1LR RBF-SVM	L1LR	L1LR
NO.2	RBF-SVM	LDA RBF-SVM		LDA RBF-SVM	LDA	LDA	
NO.3	LDA	–		–	–	RBF-SVM	

*CA, classification accuracy; AUC, area under ROC curves; SE, sensitivity; SP, specificity; CT, computational time; n.s: nonsignificant*.

Compared with the othe two classifiers, the LDA classifier showed a relatively poor generalization, which might be the reason why EEG was a nonstationary signal (Qin and Ji, 2004), and the differences between two types of signals (namely positives and negatives) could not be simply distinguished by linear mapping. The basic idea of LDA was to linearly project the multidimensional data into the feature space where two types of data could be best distinguished and to eventually create linear boundaries for the two classes. Therefore, LDA might be mainly suitable for the situation that the features of the two classes were linearly separable but not suitable for nonlinear ERP features (Liong and Foo, 2013). Moreover, LDA and RBF-SVM might also be subjected to overfitting, focusing too much on adjusting the boundary to give an optimal fit to the training set, but failing to produce a good general boundary between the two classes (Dixon and Brereton, 2009). Therefore, the cross-validation was utilized to avoid this problem in the current study. Also, the L1LR model with sparsity based on the L1-norm might avoid the overfitting problem to a certain degree (Kim et al., 2007; Park and Hastie, 2007). Moreover, the computational complexity decreased because of the sparsity of L1LR model. In summary, L1LR classifiers showed a good generalization, while LDA showed a relatively poor generalization for the emotional classification with single-trial N170 as a feature in the present study.

## Limitations

Previous studies demonstrated that the vertex positive potential (VPP) component recorded at the Fz electrode might be the positive counterpart of N170 (George et al., 1996; Itier and Taylor, 2004). Therefore, we did not discuss the VPP component in the current study. Based on the standard analysis of ERPs, we found that there was no obvious difference between N170 trials with negative emotion and N170 trials with neutral emotion after performing the paired *t*-test. Moreover, according to the behavioral analysis, there was no obvious difference in RT between positive face discrimination and neutral face discrimination. For ACC, there was no significant difference between neutral faces identification and negative faces identification. In addition, there was no significant difference on ACC between neutral faces identification and positive faces identification. The reason for this situation might be that some subjects mistakenly regarded expressionless pictures (namely neutral facial expressions) as facial pictures with negative emotions during the experiment, while some other subjects might mistakenly regard neutral facial expressions as positive expressions. Thus, we simply did the pattern classification between positive samples and negative samples. The research that included both amplitude and latency as features to classify emotions could be interesting and valuable, which might increase the classification performance. In future studies, we could continue our study from this respect and might find something interesting.

## Conclusions

In the current study, we proposed using a single-trial N170 as a feature applied in the emotional classification. The results illustrated that three classifiers, namely L1LR, RBF-SVM, and LDA, were utilized to successfully classify positive and negative samples, and L1LR showed a relatively good generalization for pattern classification of different emotions while LDA showed a relatively poor classification performance. The current study could provide beneficial information for researchers in emotion regulation; furthermore, the single-trial N170 could be a biomarker to monitor the emotional states of subjects for the BCI domain.

## Author contributions

YT Conceived, designed the experiments, and wrote the manuscript. HZ performed the experiments, analyzed the data, and wrote the first draft. YP and JL contributed reagents, materials, and analysis tools.

### Conflict of interest statement

The authors declare that the research was conducted in the absence of any commercial or financial relationships that could be construed as a potential conflict of interest.

## References

[B1] Ayalew L. Yamagishi H. (2005). The application of GIS-based logistic regression for landslide susceptibility mapping in the Kakuda-Yahiko Mountains, Central Japan. Geomorphology 65, 15–31. 10.1016/j.geomorph.2004.06.010

[B2] Bentin S. Allison T. Puce A. Perez E. McCarthy G. (1996). Electrophysiological studies of face perception in humans. J. Cogn. Neurosci. 8, 551–565. 10.1162/jocn.1996.8.6.551 20740065 PMC2927138

[B3] Blau V. C. Maurer U. Tottenham N. McCandliss B. D. (2007). The face-specific N170 component is modulated by emotional facial expression. Behav. Brain Funct. 3:7. 10.1186/1744-9081-3-7 17244356 PMC1794418

[B4] Brew C. (2016). Classifying ReachOut posts with a radial basis function SVM, in Proceedings of the Third Workshop on Computational Lingusitics and Clinical Psychology (San Diego, CA: NAACL), 138–142. 10.18653/v1/W16-0315

[B5] Caharel S. Courtay N. Bernard C. Lalonde R. Rebaï M. (2005). Familiarity and emotional expression influence an early stage of face processing: an electrophysiological study. Brain Cogn. 59:96. 10.1016/j.bandc.2005.05.005 16019117

[B6] Cai B. Xiao S. Jiang L. Wang Y. Zheng X. (2013). A rapid face recognition BCI system using single-trial ERP, in 2013 6th International IEEE/EMBS Conference on Neural Engineering (NER) (San Diego, CA: IEEE), 89–92. 10.1109/NER.2013.6695878

[B7] Chanel G. Kierkels J. J. M. Soleymani M. Pun T. (2009). Short-term emotion assessment in a recall paradigm. Int. J. Hum. Comput. Stud. 67, 607–627. 10.1016/j.ijhcs.2009.03.005

[B8] Cook R. D. Weisberg S. (2008). Logistic Regression and Generalized Linear Models. Applied Regression Including Computing and Graphics. John Wiley & Sons, Inc.

[B9] Dixon S. J. Brereton R. G. (2009). Comparison of performance of five common classifiers represented as boundary methods: Euclidean Distance to Centroids, Linear Discriminant Analysis, Quadratic Discriminant Analysis, Learning Vector Quantization and Support Vector Machines, as dependent on data structure. Chemometr. Intelligent Lab. Syst. 95, 1–17. 10.1016/j.chemolab.2008.07.010

[B10] Eimer M. Holmes A. (2007). Event-related brain potential correlates of emotional face processing. Neuropsychologia 45, 15–31. 10.1016/j.neuropsychologia.2006.04.022 16797614 PMC2383989

[B11] Eugenio B. D. Glass M. (2015). Squibs and discussions-the Kappa statistic: a second look. Comput. Linguist. 30, 95–101. 10.1162/089120104773633402

[B12] Fazel-Rezai R. Allison B. Z. Guger C. Sellers E. W. Kleih S. C. Andrea K. (2012). P300 brain computer interface: current challenges and emerging trends. Front. Neuroeng. 5:14. 10.3389/fneng.2012.00014 22822397 PMC3398470

[B13] Fox E. Lester V. Russo R. Bowles R. J. Pichler A. Dutton K. (2000). Facial expressions of emotion: are angry faces detected more efficiently? Cogn. Emot. 14, 61–92. 10.1080/026999300378996 17401453 PMC1839771

[B14] George N. Evans J. Fiori N. Davidoff J. Renault B. (1996). Brain events related to normal and moderately scrambled faces. Brain Res. Cogn. Brain Res. 4, 65–76. 10.1016/0926-6410(95)00045-3 8883920

[B15] Hinojosa J. A. Mercado F. Carretié L. (2015). N170 sensitivity to facial expression: a meta-analysis. Neurosci. Biobehav. Rev. 55, 498–509. 10.1016/j.neubiorev.2015.06.002 26067902

[B16] Hong B. Guo F. Liu T. Gao X. Gao S. (2009). N200-speller using motion-onset visual response. Clin. Neurophysiol. 120, 1658–1666. 10.1016/j.clinph.2009.06.026 19640783

[B17] Itier R. J. Taylor M. J. (2004). N170 or N1? Spatiotemporal differences between object and face processing using ERPs. Cereb. Cortex 14, 132–142. 10.1093/cercor/bhg111 14704210

[B18] Jin H. Ling C. X. (2005). Using AUC and accuracy in evaluating learning algorithms. IEEE Trans. Knowl. Data Eng. 17, 299–310. 10.1109/TKDE.2005.5027295638

[B19] Jin J. Allison B. Z. Sellers E. W. Brunner C. Horki P. Wang X.. (2011). An adaptive P300-based control system. J. Neural Eng. 8:036006. 10.1088/1741-2560/8/3/036006 21474877 PMC4429775

[B20] Keating K. A. Cherry S. (2004). Use and interpretation of logistic regression in habitat-selection studies. J. Wildlife Manage. 68, 774–789. 10.2193/0022-541X(2004)068[0774:UAIOLR]2.0.CO;2

[B21] Khushaba R. N. Wise C. Kodagoda S. Louviere J. Kahn B. E. Townsend C. (2013). Consumer neuroscience: assessing the brain response to marketing stimuli using electroencephalogram (EEG) and eye tracking. Expert Syst. Appl. 40, 3803–3812. 10.1016/j.eswa.2012.12.095

[B22] Kim S. J. Koh K. Lustig M. Boyd S. Gorinevsky D. (2007). An interior-point method for large-scale L_1_ regularized least squares. IEEE J. Select. Top. Signal Process. 1, 606–617. 10.1109/JSTSP.2007.91097127295638

[B23] Landis J. R. Koch G. G. (1977). The measurement of observer agreement for categorical data. Biometrics 33, 159–174. 10.2307/2529310 843571

[B24] Lin Y. P. Wang C. H. Wu T. L. Jeng S. K. Chen J. H. (2008). Support vector machine for EEG signal classification during listening to emotional music, in 2008 IEEE 10th Workshop on Multimedia Signal Processing (IEEE), 127–130. 10.1109/MMSP.2008.466506127295638

[B25] Lindquist K. A. Barrett L. F. (2012). A functional architecture of the human brain: emerging insights from the science of emotion. Trends Cogn. Sci. 16, 533–540. 10.1016/j.tics.2012.09.005 23036719 PMC3482298

[B26] Ling C. X. Huang J. Zhang H. (2003). AUC: a statistically consistent and more discriminating measure than accuracy, in Proceedings of the 18th International Joint Conference on Artificial Intelligence. (Mexico: Morgan Kaufmann Publishers Inc.), 519–524.

[B27] Liong C. Y. Foo S. F. (2013). Comparison of linear discriminant analysis and logistic regression for data classification[C]. Amer. Inst. Phys. 2013, 1159–1165. 10.1063/1.480126224313031

[B28] Liu X. Liao Y. Zhou L. Sun G. Li M. Zhao L. (2013). Mapping the time course of the positive classification advantage: an ERP study. Cogn. Affect. Behav. Neurosci. 13, 491–500. 10.3758/s13415-013-0158-6 23504806

[B29] Meng F. Tong K. Y. Chan S. T. Wong W. W. Lui K. H. Tang K. W.. (2008). BCI-FES training system design and implementation for rehabilitation of stroke patients, in IEEE International Joint Conference on Neural Networks (Hong Kong), 4103–4106. 10.1109/IJCNN.2008.4634388

[B30] Mühl C. Allison B. Nijholt A. Chanel G. (2014). A survey of affective brain computer interfaces: principles, state-of-the-art, and challenges. Brain Comput. Interf. 1, 66–84. 10.1080/2326263X.2014.912881

[B31] Müller K. R. Anderson C. W. Birch G. E. (2003). Linear and nonlinear methods for brain-computer interfaces. IEEE Trans. Neural Syst. Rehabil. Eng. 11, 165–169. 10.1109/TNSRE.2003.814484 12899264

[B32] Murugappan M. Rizon M. Nagarajan R. Yaacob S. Hazry D. Zunaidi I. (2008). Time-frequency analysis of EEG signals for human emotion detection, in 4th Kuala Lumpur International Conference on Biomedical Engineering 2008 (Kuala Lumpur).

[B33] Negro F. Muceli S. Castronovo A. M. Holobar A. Farina D. (2016). Multi-channel intramuscular and surface EMG decomposition by convolutive blind source separation. J. Neural Eng. 13, 26–27. 10.1088/1741-2560/13/2/026027 26924829

[B34] Netherlands S. (2008). Support Vector Machine (SVM), in Encyclopedia of Genetics, Genomics, Proteomics and Informatics (Dordrecht: Springer Netherlands), 1901.

[B35] Panicker R. C. Puthusserypady S. Sun Y. (2011). An asynchronous P300 BCI with SSVEP-based control state detection. IEEE Trans. Biomed. Eng. 58, 1781–1788. 10.1109/TBME.2011.2116018 21335304

[B36] Park M. Y. Hastie T. (2007). *L*_1_-regularization path algorithm for generalized linear models. J. R. Stat. Soc. B Stat. Methodol. 69, 659–677. 10.1111/j.1467-9868.2007.00607.x24843434

[B37] Pfurtscheller G. Neuper C. (2001). Motor imagery and direct brain-computer communication. Proc. IEEE 89, 1123–1134. 10.1109/5.93982927295638

[B38] Provost F. Fawcett T. Kohavi R. (1997). The case against accuracy estimation for comparing induction algorithms, in Proceedings of the Fifteenth International Conference on Machine Learning, 445–453.

[B39] Qin S. Ji Z. (2004). Extraction of features in EEG signals with the non-stationary signal analysis technology, in International Conference of the IEEE Engineering in Medicine and Biology Society (San Francisco, CA), 349. 10.1109/IEMBS.2004.1403164 17271682

[B40] Rellecke J. Sommer W. Schacht A. (2013). Emotion effects on the n170: a question of reference? Brain Topogr. 26, 62–71. 10.1007/s10548-012-0261-y 23053603

[B41] Schupp H. T. Ohman A. Junghöfer M. Weike A. I. Stockburger J. Hamm A. O. (2004). The facilitated processing of threatening faces: an ERP analysis. Emotion 4:189. 10.1037/1528-3542.4.2.189 15222855

[B42] Sharma A. Paliwal K. K. (2015). Linear discriminant analysis for the small sample size problem: an overview. Int. J. Machine Learn. Cybernet. 6, 443–454. 10.1007/s13042-013-0226-926811110

[B43] Smith M. L. Gosselin F. Schyns P. G. (2012). Measuring internal representations from behavioral and brain data. Curr. Biol. 22, 191–196. 10.1016/j.cub.2011.11.061 22264608

[B44] Song J. Liu M. Yao S. Yan Y. Ding H. Yan T.. (2017). Classification of emotional expressions is affected by inversion: behavioral and electrophysiological evidence. Front. Behav. Neurosci. 11:21. 10.3389/fnbeh.2017.00021 28232793 PMC5298963

[B45] Sourina O. Liu Y. Nguyen M. K. (2012). Real-time EEG-based emotion recognition for music therapy. J. Multimodal User Interfaces 5, 27–35. 10.1007/s12193-011-0080-626811110

[B46] Tian Y. Xu W. Zhang H. Tam K. Y. Zhang H. Yang L.. (2018). The scalp time-varying networks of N170: reference, latency, and information flow. Front. Neurosci. 12:250. 10.3389/fnins.2018.00250 29720933 PMC5915542

[B47] Tian Y. Yao D. (2013). Why do we need to use a zero reference? Reference influences on the ERPs of audiovisual effects. Psychophysiology 50, 1282–1290. 10.1111/psyp.12130 23941085

[B48] Toshiro F. Eiichi O. Kouzou T. Atsushi K. Hiroaki T. Toshiaki O.. (2012). Changes in event-related desynchronization and synchronization during the auditory oddball task in schizophrenia patients. Open Neuroimag. J. 6, 26–36. 10.2174/1874440001206010026 22870167 PMC3409351

[B49] Yang P. Liu Q. Metaxas D. N. (2009). Boosting encoded dynamic features for facial expression recognition. Pattern Recognit. Lett. 30, 132–139. 10.1016/j.patrec.2008.03.014

[B50] Yao D. (2001). A method to standardize a reference of scalp EEG recordings to a point at infinity. Physiol. Meas. 22, 693–711. 10.1088/0967-3334/22/4/305 11761077

[B51] Zhang D. Song H. Xu H. Wu W. Gao S. Hong B. (2012). An N200 speller integrating the spatial profile for the detection of the non-control state. J. Neural Eng. 9:026016. 10.1088/1741-2560/9/2/026016 22414615

[B52] Zhang Y. Zhao Q. Jin J. Wang X. Cichocki A. (2012). A novel BCI based on ERP components sensitive to configural processing of human faces. J. Neural Eng. 9:026018. 10.1088/1741-2560/9/2/026018 22414683

[B53] Zhu J. Hastie T. (2004). Classification of gene microarrays by penalized logistic regression. Biostatistics 5, 427–443. 10.1093/biostatistics/kxg046 15208204

