# Supplementary material for: Classification for Single-Trial N170 During Responding to Facial Picture With Emotion
Source: Front Comput Neurosci. 2018 Sep 13;12:68. doi: 10.3389/fncom.2018.00068 (PMC6146201; doi:10.3389/fncom.2018.00068)
Supplement: Supplementary file 1 [file Table_1.DOCX]

**APPENDIX 1**

Before the EEG recording experiment, 50 subjects took part in rating the scales (including emotional valence and arousal) to ensure that the emotion stimulus utilized in the current study was reasonable. Emotional valence was set to range from -3 to 3 (i.e. -3 -2 -1 0 1 2 3). 0 denoted neutral emotion, -1 represented weak negative emotion and -3 denoted strong negative emotion. Similarly, 1 represented weak positive emotion and 3 denoted strong positive emotion. After doing the statistical analysis of subjects’ valence, positive emotional valence was 1.66 ± 0.33 (mean ± SD), negative emotional valence was -1.74±0.38, and neutral emotional valence was 0.08 ± 0.17, as the Figure S1 illustrated. Arousal was set to range from 1 to 5, which denoted the degree of emotional activation. 1 denoted the weakest arousal and 5 denoted the strongest arousal.

Emotional valence and arousal were analyzed using a one-way analysis of variance (ANOVA), with emotion (positive, neutral, and negative) as the within-subjects factor. There was a significant main effect of emotion on valence (*F* = 1524.8, *p* < 0.001). Post-hoc test showed that positive emotional valence was significantly different from neutral emotional valence (*t* = 31.5, *p* < 0.001) and negative emotional valence (*t* = 38.1, *p* < 0.001). Moreover, neutral emotional valence was significantly different from negative emotional valence (*t* = 34.5, *p* < 0.001).

For emotional arousal, there was a significant main effect of emotion (*F* = 48.6, *p* < 0.001). Post-hoc test showed that positive emotional arousal was significantly different from neutral emotional arousal (*t* = 9.87, *p* < 0.001) and negative emotional arousal (*t* = 8.08, *p* < 0.001). Moreover, neutral emotional arousal was significantly different from negative emotional arousal (*t* = 12.82, *p* < 0.001).

RT was analyzed by 2 (gender: male vs. female) × 3 (expression: positive vs. negative vs. neutral) repeated-measures ANOVA. There was a significant main effect of expression (*F* = 17.77, *p* < 0.001), while non-significant main effect of gender was found (*F* = 0.06, *p* > 0.05). There was non-significant interaction between gender and expression (*F* = 0.01, *p* > 0.05). Figure S2 illustrated RT of different gender for three emotions.

Compared with other electrodes, we found the larger N170 waves at the twelve electrodes (electrodes P3, P4, P5, P6, P7, P8, PO3, PO4, PO5, PO6, PO7, and PO8). Figure S3 showed the topological energy of N170 ERPs for the whole brain and the higher energy at the twelve electrodes.

The latency of the N170 was analyzed using one-way ANOVA, with emotion (positive, neutral, and negative) as the within-subjects factor. For latency of the N170 with different emotions, different electrodes showed different results. For instance, Figure S4 illustrated the latency of the N170 with three emotions at electrode P7. The results showed a significant main effect of emotion on N170 latency (*F* = 19.77, *p* < 0.001). Moreover, post-hoc t-test demonstrated that there was no significant difference on N170 latency between positive and neutral emotion (*t* = 0.18, *p* > 0.05). However, N170 latency of negative emotion was longer than that of positive emotion (*t* = 6.32, *p* < 0.001) and neutral emotion (*t* = 6.25, *p* < 0.001).

Table S1 showed the grade point average (GPA) of subjects. The mean GPA of 20 subjects was 2.829 and the standard deviation of GPA was 0.322.


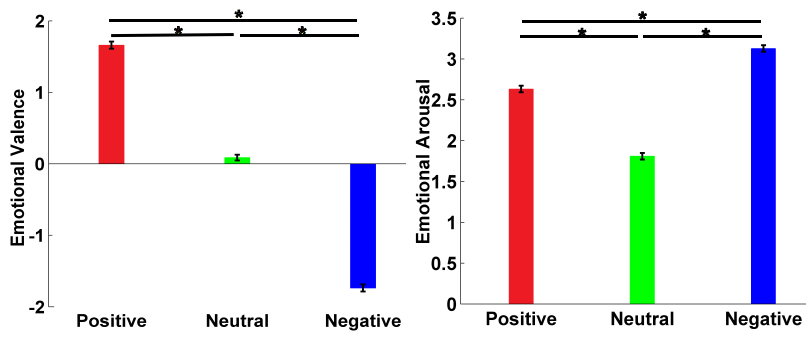


**Figure S1.** Emotional valence and arousal for three emotions. The red bar denoted positive emotion, the green bar denoted neutral emotion and the blue bar denoted negative emotion. The star denoted there was significant difference between two conditions.


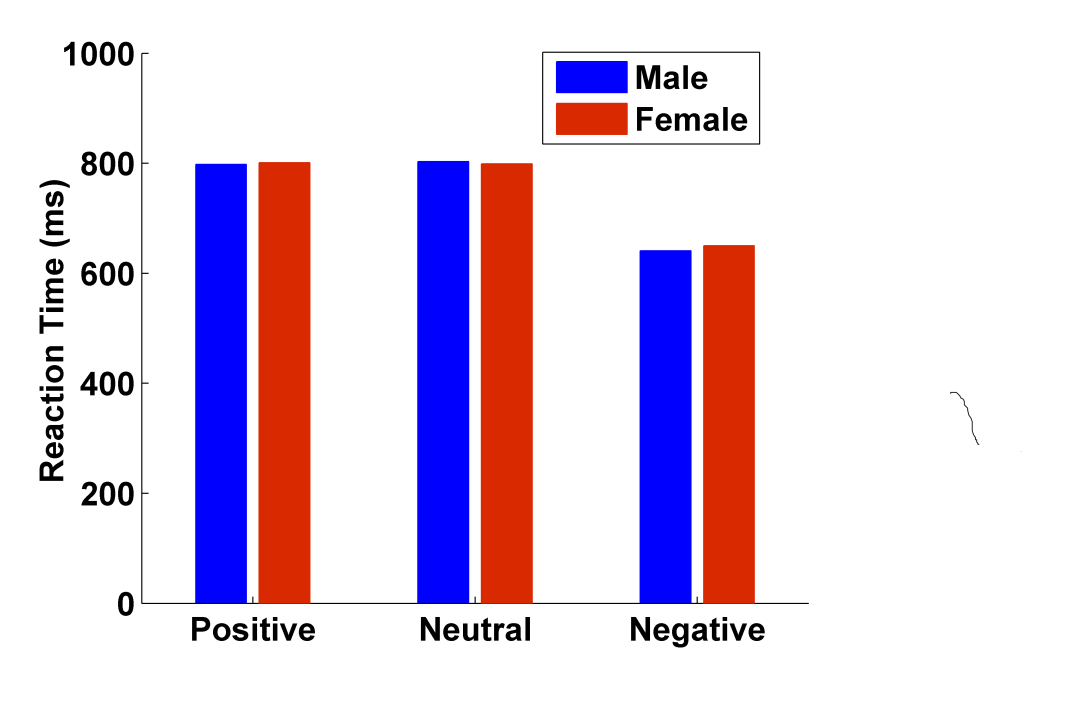


**Figure S2.** RT of different gender for three emotions.


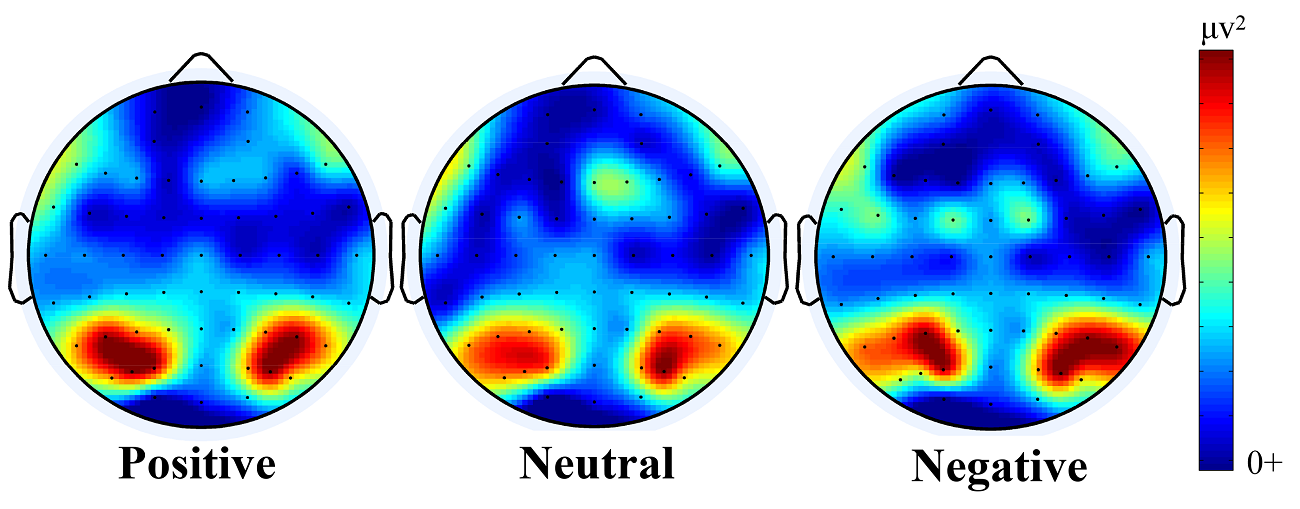


**Figure S3.** The energy of N170 ERPs with three emotions for the whole brain with topological mapping.


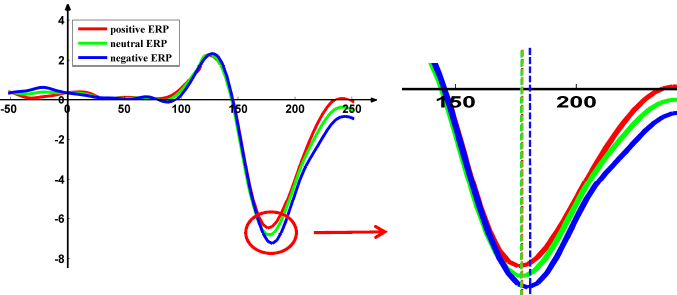


**Figure S4.** Latency of the N170 with three emotions at electrode P7.

**Table S1**. The grade point average (GPA) of subjects

| subjects | GPA |
| --- | --- |
| s1 | 2.638 |
| s2 | 3.357 |
| s3 | 3.179 |
| s4 | 2.953 |
| s5 | 2.827 |
| s6 | 2.735 |
| s7 | 3.080 |
| s8 | 3.076 |
| s9 | 3.027 |
| s10 | 3.478 |
| s11 | 2.805 |
| s12 | 2.509 |
| s13 | 2.482 |
| s14 | 2.229 |
| s15 | 2.535 |
| s16 | 2.666 |
| s17 | 2.451 |
| s18 | 2.994 |
| s19 | 2.946 |
| s20 | 2.612 |

**APPENDIX 2**

REST：Reference Electrode Standardization Technique

REST is a novel method that builds a bridge between a physical reference and the theoretical neutral reference at an infinity point. For an infinity reference, the forward EEG calculation is given by:

$V=GS$ (1)

where G is the transfer matrix referenced at infinity, only dependent on the head model, source configuration, and electrode montage; S is the source; V is the scalp EEG recording with a reference at infinity generated by S. Scalp noise is not explicitly considered in this model. For a physical reference such as the CZ referenced recordings $V_{CZ}$, we similarly have:

$V_{CZ}=G_{CZ}S$ (2)

where $G_{CZ}$ is the EEG lead-field matrix with CZ reference and $V_{CZ}$ refers to EEG scalp recordings referenced at CZ. A solution for the source distribution S is given by:

$S=G_{CZ}^{-}V_{CZ}$ (3)

where ${(G_{CZ})}^{-}$may be the Moore-Penrose generalized inverse of matrix $G_{CZ}$. From Equations 2 and 3, we can see that the source S is the same, which reflects the fact that reference choice does not influence the source localization; that is, activated neural sources in the brain are not affected by the particular reference used. The potential with reference at infinity can thus be reconstructed as the following:

$V_{REST}=G\left( G_{CZ}^{-}V_{CZ} \right)=UV_{CZ}$ (4)

where $U=GG_{CZ}^{-}$ is the final transfer matrix simultaneously determined by the lead-field matrix G and $G_{CZ}$, where G is known, and $G_{CZ}$can be easily derived from G. In addition, recordings using any other single physical electrode as reference can be mathematically transformed to the infinity reference using a formula similar to Equation 4; the only difference is the use of a specific lead-field matrix corresponding to the adopted reference.

For more details, please look at the literatures ([Yao, 2001](#_ENREF_5); [Qin et al., 2010](#_ENREF_4)) and the website: [www.neuro.uestc.edu.cn/rest](http://www.neuro.uestc.edu.cn/rest) with timely update.

**References**

Qin, Y., Xu, P., and Yao, D. (2010). A comparative study of different references for EEG default mode network: the use of the infinity reference. *Clinical Neurophysiology* 121**,** 1981-1991.

Yao, D. (2001). A method to standardize a reference of scalp EEG recordings to a point at infinity. *Physiological Measurement* 22**,** 693-711.
